# A New Method to Explore the Spectral Impact of the Piriform Fossae on the Singing Voice: Benchmarking Using MRI-Based 3D-Printed Vocal Tracts

**DOI:** 10.1371/journal.pone.0102680

**Published:** 2014-07-21

**Authors:** Bertrand Delvaux, David Howard

**Affiliations:** 1 Audiolab, Electronics Department, University of York, York, United Kingdom; 2 York Centre for Singing Science, University of York, York, United Kingdom; University of Texas Health Science Center at San Antonio, Research Imaging Institute, United States of America

## Abstract

The piriform fossae are the 2 pear-shaped cavities lateral to the laryngeal vestibule at the lower end of the vocal tract. They act acoustically as side-branches to the main tract, resulting in a spectral zero in the output of the human voice. This study investigates their spectral role by comparing numerical and experimental results of MRI-based 3D printed Vocal Tracts, for which a new experimental method (based on room acoustics) is introduced. The findings support results in the literature: the piriform fossae create a spectral trough in the region 4–5 kHz and act as formants repellents. Moreover, this study extends those results by demonstrating numerically and perceptually the impact of having large piriform fossae on the sung output.

## Introduction

**Figure 1:**
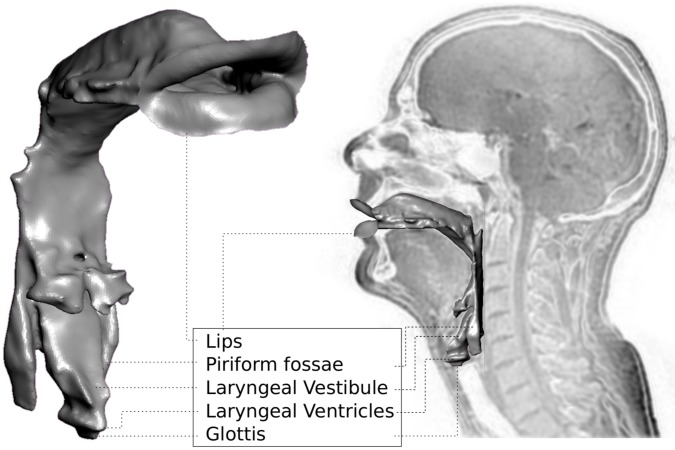
The hypopharynx cavities in the Vocal Tract. Vocal Tract profile superimposed on an MRI midsagittal slice of a singer while phonating on the vowel as in the word /st
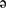
ːn/ and on a 3/4, details of the hypopharynx cavities which consist of the laryngeal vestibule, the laryngeal ventricles and the two piriform fossae, located posteriorly at the bottom of the pharynx.

The piriform fossae, or piriform sinuses, owe their name to their pear shape. This pair of bilateral cavities is located posteriorly at the bottom of the pharynx, just above the oesophageal entrance. Together with the laryngeal vestibule and ventricles, they form the hypopharyngeal cavities (see Fig 1), whose acoustic properties are thought to contribute to the acoustic uniqueness of a voice, by shaping the formants F3, F4 and F5, with large inter-speaker variations and small intra-speaker (i.e., inter-phoneme) variations [1]. In particular, the piriform fossae, as side branches of the Vocal Tract (VT) produce troughs in the region of 4 to 5 kHz [2], and play a significant role in the singer's formant between 2 and 3 kHz [3]. The singer's formant cluster is a well-established feature of the acoustic output from the VT of trained opera singers that is independent of the vowel being sung [3]. It is commonly described as a cluster of F3, F4 and F5. This suggests that the singer's formant cluster is related to a region of the VT that does not change greatly in shape with vowel articulation; anatomically, this relates to the hypopharyngeal cavities [1]. More precisely, the epilarynx (laryngeal vestibule and laryngeal ventricles) does not change greatly in shape across vowels whereas Painter [4] claims that if the volume of the piriform fossae cannot be actively enlarged, action of the inferior pharyngeal constrictor muscles, posteroanterior expansion of the epilarynx, or raising the larynx can actively reduce their volume.

Davies et al. [5] found a decrease of around 5% in F1, F2 for the vowel /a/ when the fossae were incorporated in the vocal tract as side branches. Titze and Story [6] found that the formant frequencies are slightly shifted when appending the piriform fossae to the main tract. In particular, they qualify the fossae as a formant repellent, generally pushing F1, F2, F3 and F4 lower and F5 higher.

Dang and Honda [7] carried out a study of the piriform fossae on mechanical models as well as on human subjects, injecting water in the piriform sinuses of humans phonating in a supine position and in mechanical models of the lower half of Vocal Tracts. Comparing the acoustic output with and without piriform fossae they found that the fossae behave as side branches of the main tract and have a significant effect on the transfer function. For both models and humans, they found that the epilarynx tube resonance was enhanced, and that the fossae not only affected the spectral shape in the neighbourhood of its antiresonance but also decreased the lower resonance frequencies.

Physiologically, the piriform fossae play a role in feeding: they contribute to the process of swallowing by storing temporarily a bolus of food or liquid before it is propelled into the oesophagus [8]. In some mammals (such as the wolf and the fox), it is found that the larynx directly projects into the nasopharynx, providing continuity of the airway [9] whereas the bipedal man developed a two-part pharynx (the nasopharynx and the oropharynx), which allows for the food to bypass the larynx laterally, through the piriform fossae, before swallowing [10]. Similar arrangements are found in cats, pigs, goats and the tenrec. From the evolutionary standpoint, neonates evolve an oropharyngeal anatomy comparable to that seen in the macaque (with an intranarial larynx, [10]) to the morphology shown in Fig 1.

In the present study, we investigate the spectral role of the piriform fossae by numerical simulations using the Finite Element Method and direct experimental measurements of 3D-printed full MRI-based Vocal Tracts, in contrast to the half VTs employed by Dang and Honda [7]. We introduce a new approach (inspired by a method used in room acoustics [11]) to measure the transfer function of MRI-based Vocal Tracts replicas moulded with a 3D rapid prototyping technique. We compare the experimental results with numerical simulations using the Finite Element Method. We explore the spectral differences in relation to length and volume measurements of the piriform fossae of 3 professional singers, based on MRI data. Finally, we assess perceptually the impact of having large piriform fossae on the sung output via a listening test.

## Materials and Methods

### Ethics statement

This study, labelled “MRI Capture of the Vocal Tract” (Project ID: P1135), was ethically approved by the Research Ethics Committee of the York Neuroimaging Centre. The participants provided their written consent to take part in this study.

### Singers

**Figure 2:**
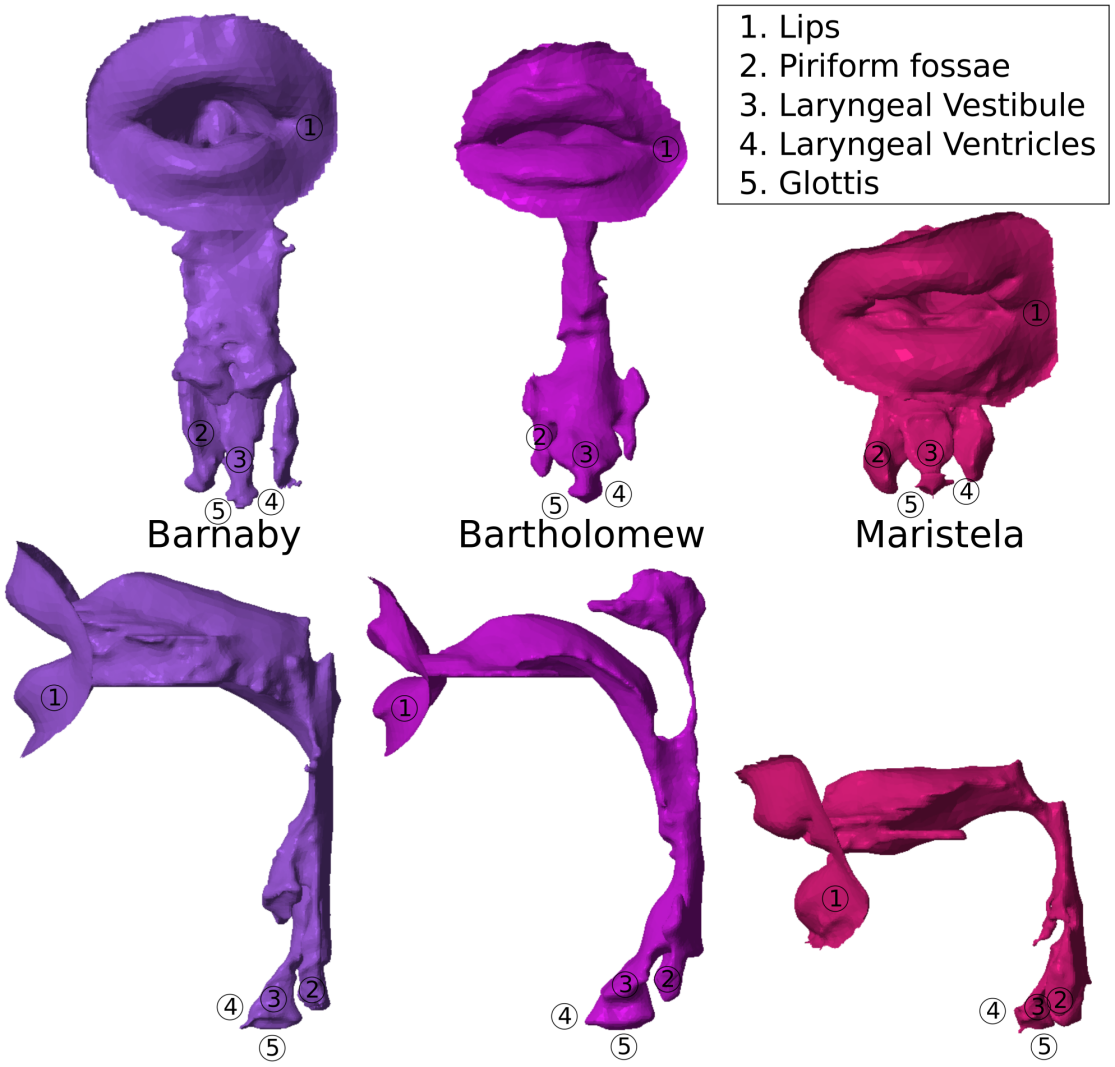
Singers from different voice categories. Scaled Vocal Tracts of 3 professional singers: from left to right, a Bass-Baritone (Barnaby), a Bari-Tenor (Bartholomew), and a Mezzo-Soprano (Maristela).

For this study, 3 professional singers sang in an MRI scanner, in a supine position (see Fig 2 for their MRI-based Vocal Tracts). The corpus is composed of 1 Mezzo-Soprano, 1 Bari-Tenor and 1 Bass-Baritone. In order to retain anonymity, but to remind the reader what voice type the singers belong to, each singer has been assigned a name with mnemonic similarity to their voice type as follows:

- **BarnaBy** is a **B**ass-**B**aritone, aged 31.
- **BarTholomew** is a **B**ari-**T**enor, aged 34.
- **MariStela** is a **M**ezzo-**S**oprano, aged 29.

These professional singers have extensive experience performing in famous Opera houses including La Scala Milan, Deutsche Opern Berlin, Covent Garden London, English National Opera London, Opera Comique Paris and La Monnaie Brussels. Further details about the singers are referred on Table 1.

**Table 1:** Singers' data.

	Age	Classification	Fach	Range	Pitch	Token(s)
Maristela	29	National	Mezzo-Soprano	F3-C#6	C4	/hαːd/
Bartholomew	34	International	Bari-Tenor	E2-D5 (G5)	F#3	/hαːd/
Barnaby	31	National	Bass-Baritone	C2-A4 (A5)	G#2	/hαːd/, /p 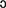 ːt/, /st 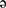 ːn/, /fuːd/, /niːp/

Age, classification according to the Bunch and Chapman criteria [30], Fach, range, sung pitch (between brackets for falsetto), token(s) sung for the 3 professional singers.

The scan of the 3 professional singers have been acquired according to the protocol described in [12]. They were tasked to choose a pitch on which they can comfortably sustain a moderately loud phonation on a phoneme (see Table 1) during the acquisition time (approximately 16 s) and were instructed to then attempt maintaining the articulatory setting in an unvoiced condition through breathing for the remainder of the scan, in case of breathlessness. No instruction was given regarding the operatic quality of voice to be produced. Scans are made at the York Neuroimaging Centre (YNiC), using a General Electric 3.0 T HDx Excite MRI Scanner. The scan developed was a 3D fast gradient echo sequence, [13]: *The relaxation time was 4.8 ms and the excitation time was 1.7 ms. Acquisition is isotropic 2mm in a* 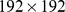 *matrix. Output is then interpolated to* 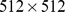 *using 50% slice overlap giving an effective anisotropic output of* 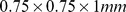 *. A stack of 80 images is produced in the midsagittal plane in approximately 16 s.*

A consideration to account for, when using magnetic resonance imaging to scan the human head is how different might be the phonation between the supine and standing positions. Gravity is thought to affect the articulations, resulting in a backwards movement of the tongue and a subsequent narrowing of the pharynx [14], [15]. Nevertheless, the phonetic effect of a supine phonation are thought to be minimal, perhaps with the aid of compensatory articulations [15], [16]. The tip of the tongue has been observed to be subject to a significant retraction in the case of a sustained supine phonation, resulting in artifacting motion in the images [17]. To prevent from such image alterations, the subjects were tasked to consider carefully the tongue position during phonation. Note that Speed [13] has recorded the subjects in a supine/standing position in a 6-sided anechoic chamber before and after the supine phonation in the MRI scanner and found that there is a spectral consistency between the supine and standing phonation, despite the gravitational pull on the abdomen during phonation.

Maintaining a constant vocal tract configuration during phonation is crucial to prevent motion artifacting in the image [12]: alterations in the stability of phonation can arise from gravity, lung volume, required longevity of sound and fatigue [17]. According to [13], the data of Barnaby were acquired during the most stable phonation which defines the clearest edges between the structures on the MRI images, leading to the most accurate segmentation of the MRI data. This is the is the reason why the data of Barnaby were chosen to compare his VT configuration singing on different vowels, as in the words /hαːd/, /p
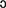
ːt/, /st
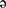
ːn/, /fuːd/ and /niːp/. Out of these 5 MRI-based VTs, /st
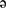
ːn/, /fuːd/ and /p
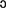
ːt/ were also 3D-printed to enable comparisons between numerical simulations and experimental measurements.

### MRI-based 3D-printed Vocal Tracts

The VT models (VTMs) were moulded based on volumetric MRI data collected while Barnaby was singing 3 English vowels in a supine position [12], by a 3D fast gradient echo sequence.

The MRI data were then segmented with the open source code ITK-Snap, to rebuild a 3D Vocal Tract, whose .STL file was then sent for 3D rapid prototyping. The material used was VeroWhitePlus Opaque. The tracts were printed on an Object24 3D Printer.

The vocal tracts can be opened just above the valleculae to enable plasticine to be placed in the cavities. The thickness of the shell of the VT is 2 mm.

### Experimental Set-up

**Figure 3:**
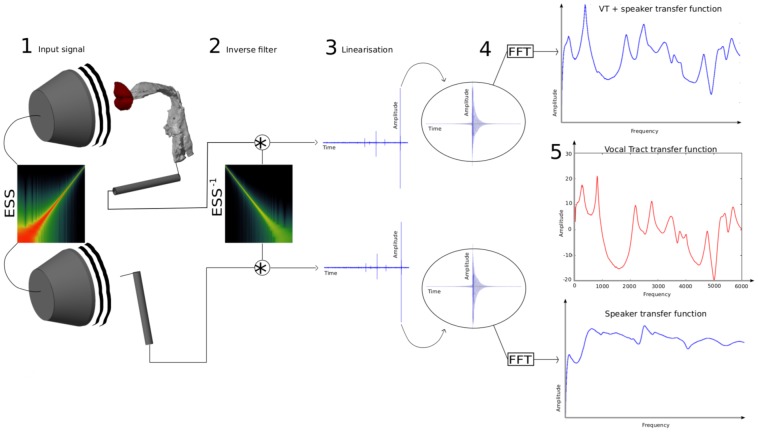
Overview of the experimental method. An Exponential Sine Sweep (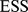) is given as an input signal to the driver (1). The output recorded via a probe microphone is convolved with the inverse filter (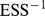) (2). It results in a temporal separation of the Linear Impulse Response (LIR) and the harmonic distortions (3). An FFT is performed on the LIR to give the linear transfer function of the system (4). Processes 1 to 4 are repeated twice: once with the Vocal Tract, and once without. Both spectra are then subtracted (logarithmic vertical scale) to give the transfer function of the standalone Vocal Tract.

A new experimental method is used to measure the impulse response and hence the transfer function of the MRI-based 3D-printed Vocal Tracts. The method is based on Farina's methodology [11] to measure simultaneously the linear response and harmonic distortions of a room with an exponential sine sweep, 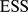. Fig 3 overviews the method which is developed in the following subsections:

1. The driver is given an input signal (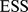) which is recorded via a probe microphone.

2. The output of the microphone is then convolved with the inverse filter of the input signal (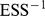).

3. As a result, the impulse response is “linearised”, i.e. the Linear Impulse Response (LIR), and the harmonic distortions are split apart.

4. An FFT is performed on the LIR, giving the transfer function.

5. The transfer function of the driver alone is subtracted from that with the VTM, giving as a final result the transfer function of the VTM, which is independent of the driver's frequency response.

NB: here, processes 2+3 are termed “Linearisation of the impulse response”

Processes 1 to 4 in Fig 3 are operated twice: once with the VTM, and once without. The resulting spectra are subtracted from one another (5 in Fig 3) to provide the transfer function of the VTM.

The experiment was carried out in a 6-sided anechoic chamber, at the temperature of 5°C. A G.R.A.S. probe microphone type 40SA was used at the glottis end. The signal was preamplified by a power Module type 12AA before being written on a USB type device (TASCAM) at a 192 kHz sampling rate and at 24 bits resolution as a WAV file. The driver was situated at 3 cm from the lips end.

#### Exponential Sine Sweep

An exponential sine sweep (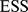) is of the form 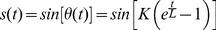
(1)where 
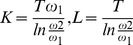
(2)with 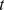 being the time, 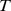 the duration of the sweep and 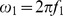 and 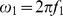 the lower and higher extremities of the frequency range swept by the sine. This signal exhibits a -3dB/octave slope [11].

#### Linearisation of the impulse response

Let 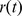 be the room/cavity response to the excitation signal 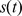 defined in (1). The room/cavity impulse response 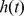 can be extracted by convolving 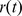 with the inverse filter of 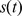 [11], [18], [19]. The exponential sweep (which is a causal signal) is temporally reversed and then delayed to obtain a causal system [20]. However, if we time-reverse the excitation signal 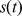, it still exhibits a 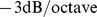. Therefore, we need to compensate this energy drop by modulating the amplitude of the time-reversed signal with a 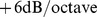 envelope so that the inverse filter exhibits a 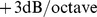 slope [11], [19]. We create an inverse filter 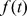, which, after being convolved with the system response, yields to the impulse response. 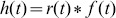
(3)This is termed post-modulation, in opposition to a pre-modulation suggested by [19], which modulates the input signal directly so that it has a flat spectrum and the reversed-time signal exhibits a flat spectrum alike. The post-modulation term which is to be multiplied with the time-reversed input signal is of the form [19]: 
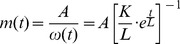
(4)where 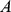 is a scalar representing the modulation amplitude. At time 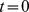, the instantenous frequency 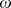 equals 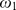. In this condition, we can solve for A in (4), assuming arbitrarily that 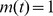 at 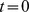: 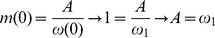
Substituting A in (4) gives 
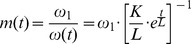



Modulating the time-reversed signal gives: 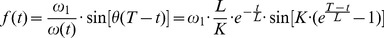
(5)which exhibits a slope of 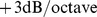.

Having designed an inverse filter which counter-balances the 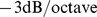 slope, it is convolved with the system response. The convolution results in a series of impulse responses, separated on the time axis. As can be seen on Fig S1 (A), the Linear Impulse Response (LIR) of the system and its harmonic distortions are temporally separated. Hence, access can be gained simultaneously to the LIR itself and the impulse response of any harmonic distortion.

*Note about the harmonic distortions*

Electro-mechanical transducers, such as those used in speakers and microphones, are non-linear systems, i.e. they do not react proportionally to the input signal given. In addition to the linear response of the system, such transducers resonate at several frequencies, the *harmonic distortions* of the device. The method described herein allows access to the linear response deprived from the harmonic distortions generated in both the speaker and the microphone. Therefore, this method is essentially independent of the speaker and the microphone (see Fig S1 (C)).

The convolution packs the harmonic distortions before the linear response on the time axis, as can be seen on Fig S1 (B). The linear response is located at the time 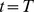 and the harmonic distortions are parallel to it.

The big improvement of the method developed in [11] resides in the fact that applying a Fast Fourier Transform (FFT) to the Linear Impulse Response removes the inherent harmonic distortions on the transfer function of the system.

#### Fast Fourier Transform

Each impulse response, starting with the LIR, is manually isolated from the other impulse responses and an FFT is performed on it, leading to the linear transfer function of the system. Fig S1 (D) shows the transfer function of the harmonic distortions and the linear response.

To isolate the LIR, Audacity software was used to zoom onto a window encompassing only the linear response, (the amplitude being switched to a logarithmic scale to assess more accurately the time interval between the start and the end of the impulse response).

To perform the FFT, an algorithm was used on each impulse response. For a time duration 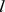:

1. **Find the next power of 2**

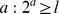



2. **Normalisation**

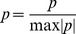



3. **Zero-padding**

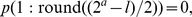


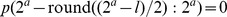



4. **FFT**

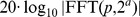
as shown in Fig S2 (C). This process is realised on 3 impulse responses from the VTM and complex-averaged in order to remove the inherent noise.

*Transfer function*

We need to perform the processes 1 to 4 (in Fig 3) twice, once to obtain the transfer function of the VTM, and again to obtain the transfer function of the driver alone. We can then subtract both spectra to get the transfer function of the VT model. The method is driver-independent.

*Note about* 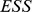 *and* 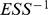


**Figure 4:**
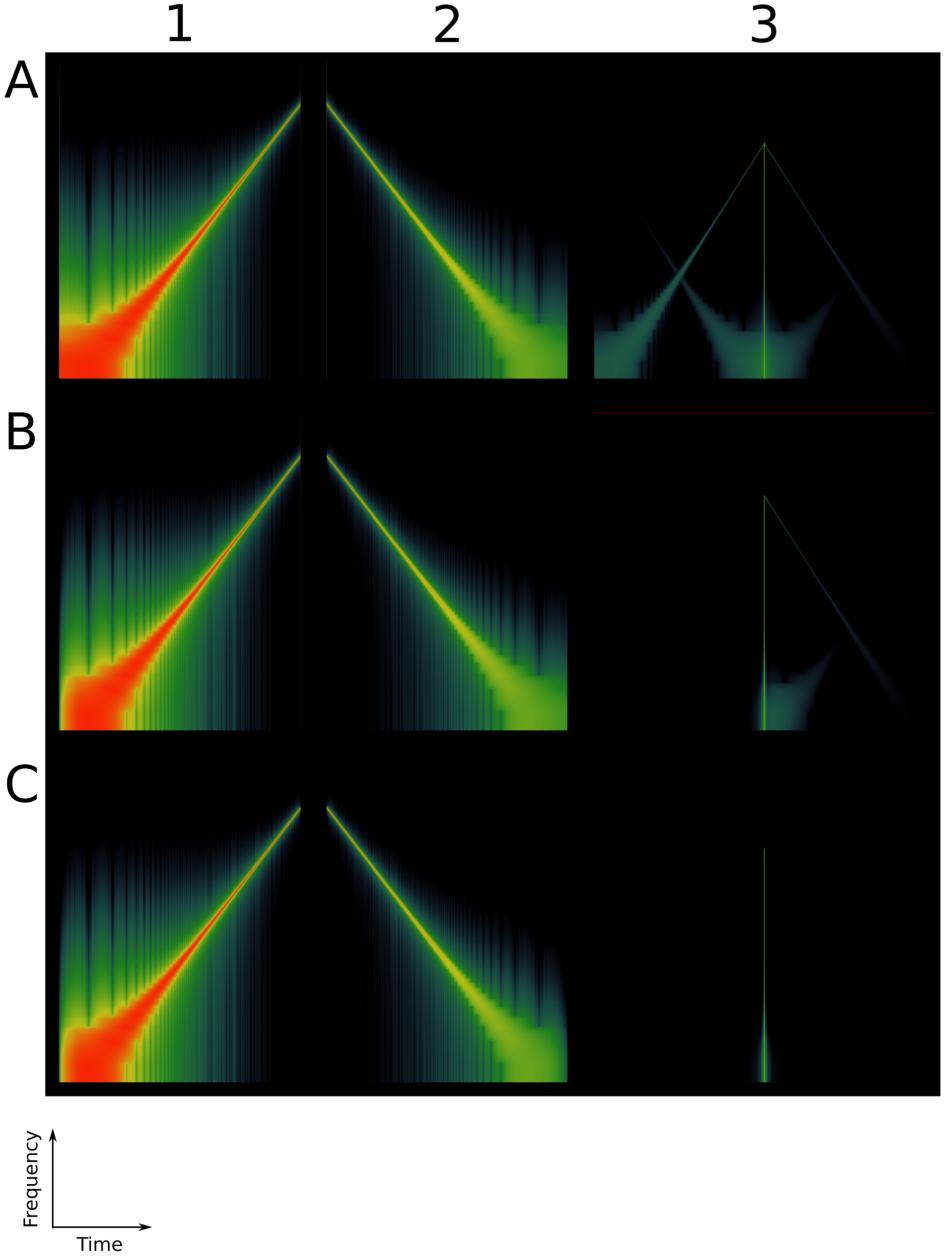
Pre- and post-envelope applied to the Exponential Sine Sweep (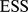). An Exponential Sine Sweep (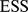) of the form (1) has a burst of energy across the whole spectrum both at its start and at its end (A1). Once convolved with its inverse filter (A2), it leads to an impulse response and its echoes in the frequency-time space (A3). Providing a smooth start to the (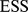) (B1), and convolving it with its inverse filter (B2) removes the pre-ringing (B3). Providing the (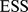) with both a smooth start and a smooth end (C1), and convolving it with its inverse filter (C2) removes both the pre- and the post-ringing (C3).

Using the 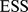 (1) as input signal, and the inverse filter (5) per se, and plotting spectrograms (frequency versus time), it can be seen that there is an instantaneous burst of energy at the start and at the end of the sweep (see the green vertical lines in Fig 4 (A1)). These are due to the fact that the sweep starts and ends non-smoothly, i.e. the slope is not continuous at the time 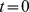 and the sweep does not necessarily cross the time axis at 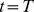. If we convolve both those signals the result is an impulse response and its echoes in the frequency-time space, as in Fig 4 (A3). The idea is to provide the sine sweep with a fade-in and a fade-out.

#### A smooth start

In Fig S2 (A), we can see that the transition at the start of the sweep is not smooth. This is due to the fact that before the sweep, the signal value is zero, with zero slope, and suddenly, at the start of the sweep, the slope abruptly changes, creating a slope discontinuity, resulting in a burst of energy across the whole spectrum, prior to the sweep.

The first derivative at the time origin gives the transition slope. The first time derivative of (1) is 
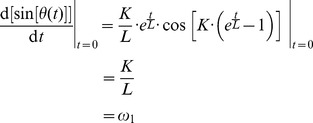
which is a non-zero slope.

To smooth this transition, the start of the signal is multiplied by a sine-squared envelope (the result is displayed in Fig S2 (A)). Being part of the sigmoid family, it ensures a smooth transition between a threshold value and a fixed value. This transition is applied between the start frequency of the sweep, 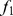 and a frequency fixed by the user, 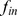. The overall algorithm is as follows:

1. Find the time at which the instantaneous frequency is equal to 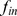.
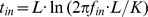



2. Find the maximum sampled time lesser than or equal to 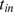.
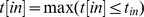



3. Generate the envelope. 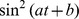



4. Multiply the signal by the envelope from 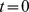 to 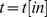.

The envelope needs to satisfy the following conditions: it ramps up from zero at frequency 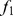 to 1 at frequency 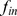, after a quarter of a period. In other words, we need to find parameters 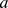 and 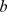 such as 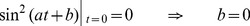
(6)

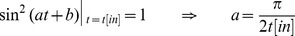
(7)Once the pre-envelope has been applied, we see that the left vertical green line (the broad-band burst of energy preceding the sweep in Fig 4 (A3)), the “pre-ringing” to quote Farina [11], disappears.

#### A smooth end

The sweep stops abruptly as soon as the frequency upper limit has been reached, and it is very unlikely that at this exact frequency the amplitude of the sine sweep would be zero (see Fig S2 (B)). For this reason, the sine sweep defined in (1) generally creates a broad-band burst of energy, occurring as it ends. A post-envelope needs to be performed to smooth down the end of the sweep unto zero. For this purpose, we apply a sine-squared function which takes the value 1 at an upper fixed frequency 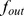 and fades out smoothly to reach zero at 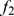.

The algorithm is as follows:

1. Find the time at which the instantaneous frequency is equal to 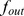.
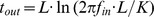



2. Find the minimum sampled time greater than or equal to 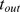.
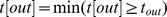



3. Generate the envelope. 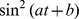



4. Multiply the signal by the envelope from 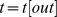 to 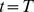.

We need to find parameters 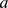 and 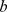 such as the sine-squared goes from the value 1 at 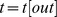 to zero at 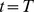 within a quarter of a period: 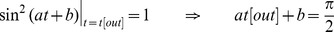
(8)

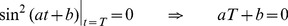
(9)


Subtracting (9) from (8) gives 
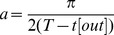
(10)


Once the pre- and post-envelope have been applied, we see that both the left and the right vertical green line (the broad-band burst of energy preceding and following the sweep respectively), the “pre-ringing” and the “post-ringing” [11] disappear as shown on Fig 4 (C3).

### Numerical method

The software ACTRAN (www.fft.be) was used to perform simulation of the transfer functions of the MRI-based Vocal Tracts with the Finite Element Method, implementing the wave equation 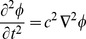 on the Vocal Tract. A point source is used as excitation at the glottis end and a probe microphone situated 3 cm far from the lips end records the pressure versus the frequency, to obtain the transfer function. A frequency independent absorption factor is set to the value 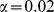 for the walls of the Vocal Tract. The absorption factor 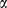 is defined as 
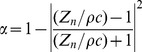
(11)where 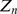 is the normal acoustic impedance, 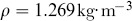 the air density and 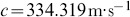 the speed of sound to meet the experimental conditions of the anechoic chamber.

### Listening Test

The sound outputs of 3 singers (Barnaby, Bartholomew and Maristela) were recorded in a 6-sided anechoic chamber, in a supine position matching phonation position in the MRI scanner. The subjects were fitted with a headset mounted AKG CK77 omnidirectional lavalier microphone and a set of Audio-Technica ATH-M30 closed-back headphones [12].

The listening test constituted 6 pairs of sounds, where each pair comprises a specific sung vowel with and without the piriform fossae. To create the version without piriform fossae, the sound outputs of the singers with piriform fossae were filtered to mimic how the singers would have sounded without piriform fossae. The filter subtracts the spectrum with piriform fossae from that without piriform fossae. For each pair of sounds (i.e. with and without piriform fossae), 10 expert listeners were asked the question “Which one would you qualify as a resonant voice?” after begin instructed with the definition of a “resonant voice” as: *a voice production that is both easy to produce and vibrant in the facial tissues* [21]. They were given the following choices as answers: first sound, second sound or no preference.

## Results

The first subsection benchmarks the new experimental method against theoretical predictions and numerical simulations of the acoustic modes of a cylinder, the second compares the experimental and numerical results of MRI-based Vocal Tracts and the last one assesses the spectral impact of the piriform fossae on the human singing voice.

### Benchmarking

Let us first examine a tube with a uniform cross-section. The modes of an open-closed cylinder are of the form: 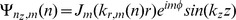
with the eigenvalues 
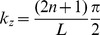
(12)giving the name of a *quarter wavelength resonator*, where 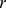, 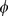 and 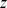 are the cylindrical coordinates and 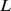 is the length of the tube.

**Figure 5:**
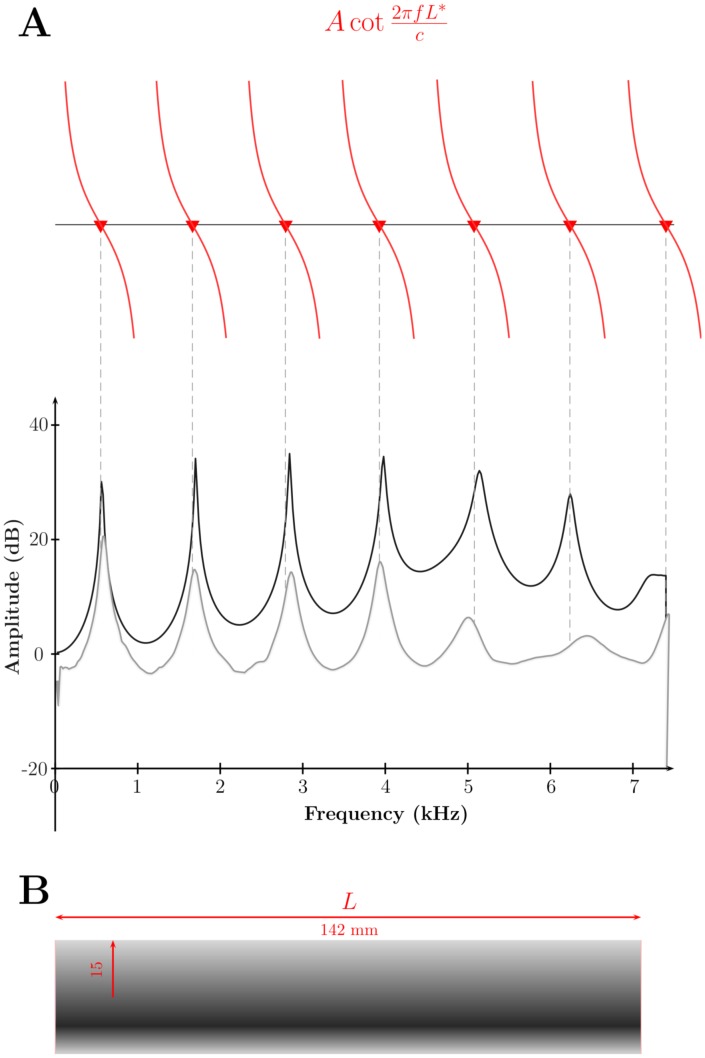
Tube resonances (theoretical predictions, numerical results, experimental measurements). The resonances of a cylinder closed at one end and opened at the other end are given as theoretical predictions (red triangles and dashed lines), numerical simulations (in black) and experimental measurements (in grey).

When its length is large in comparison with the wavelength, the resonant frequencies can be approximated under the 1D assumption of plane wave propagation [22]; the cross-sectional dimension of the tubes should be less than a half-wavelength, which means it is valid up until about 5 kHz (here, the diameter of the cylinder is 30 mm, see next paragraph). Under this assumption, the acoustic modes are given [3], [22] as the solutions of 
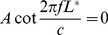
(13)where 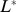 is the acoustical length of the tube (see below), 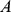 its cross-section, 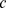 the speed of sound and 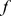 the frequency. The resonances of one tube of dimensions: Length = 142 mm, Radius = 15 mm, Flange = 2 mm are displayed in Fig 5. The theoretical predictions are the roots of equation (13), plotted in red. These are linked to the numerical simulations (FEM) in black and experimental results in grey by the dotted lines. The experimental and numerical results agree with the theoretical predictions of the modes of the cylinder.

#### Acoustical length

The length 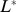 used in (13) is the effective acoustical length of the tube, i.e. the physical length plus the end correction which accounts for the small volume of air outside the tube vibrating along with the air inside [23]. The end correction factor is known analytically for two extreme cases, i.e. a cylinder with a circular flange of infinite and zero dimensions [24], [25]. The length correction for low frequencies in these two cases is 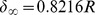 and 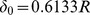, where 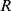 is the radius of the cylinder. A fit formula for an infinite flange is given by Dalmont et al. [26] after Norris and Cheng (1989) for 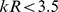: 
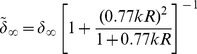
(14)where 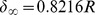, 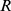 is the radius of the inner tube and 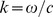 is the wavenumber.

#### OECC

The Open End Correction Coefficient (OECC) is the coefficient by which 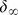 has to be multiplied to account for the finiteness of the flange bearing in mind that the end correction factor is only known analytically for 2 extreme cases; a cylinder with a circular flange of infinite and zero dimensions. Based on experimental data, Dang et al. [27] after Hall (1987) give the following empirical formula describing the relation between the OECC and the width of the flange for a low-frequency approximation, 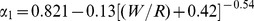
(15)where 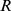 is the radius of the open end and 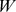 is the width of the flange.

### Experimental versus Numerical

**Figure 6:**
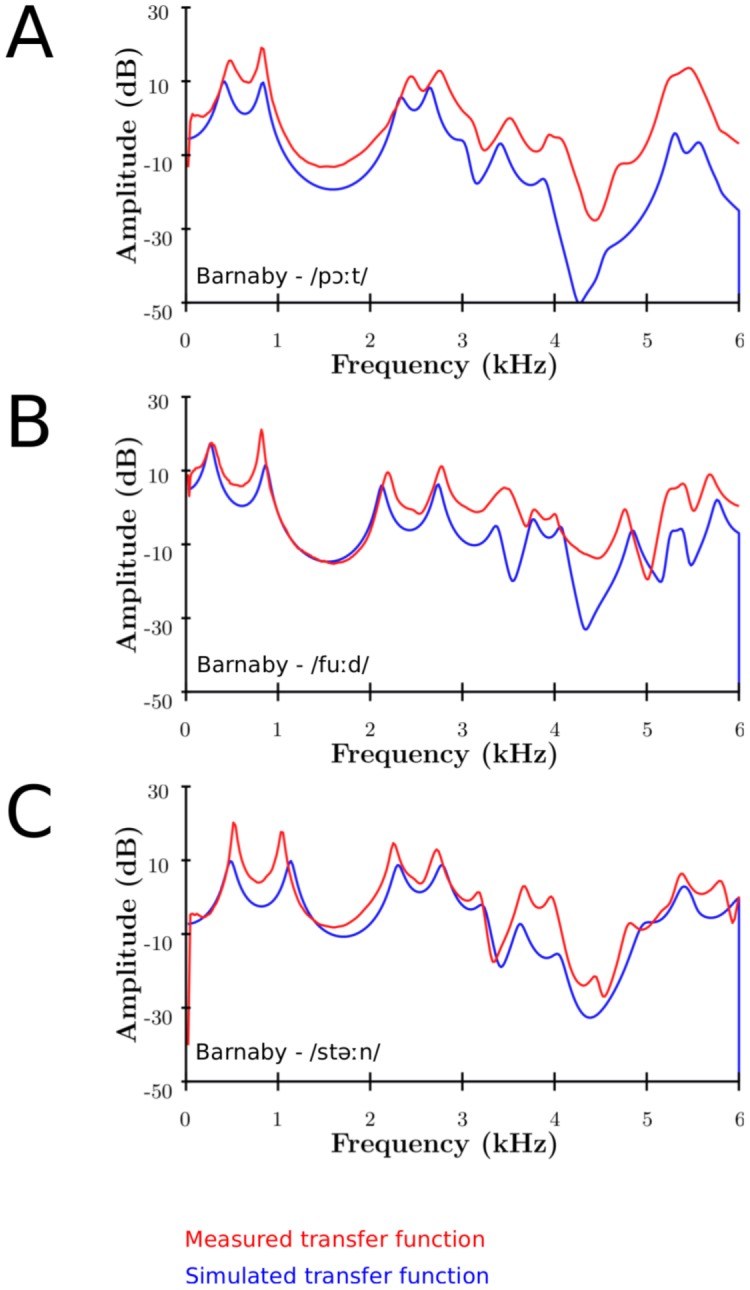
Numerical versus Experimental. Measured (in red) and simulated (in red) transfer functions of MRI-based Vocal Tracts of Barnaby singing on the vowels as in /p
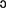
ːt/, /fuːd/ and /st
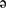
ːn/.

Fig 6 (A, B, C) shows the comparison between numerical simulations (in blue) and experimental results (in red) for Barnaby phonating on /p
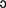
ːt/, /fuːd/ and /st
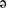
ːn/ respectively. The numerical simulations are in good agreement with the experimental measurements, although there are discrepancies, mostly due to the fact that the simulation propagates a lossless wave equation ignoring actual Vocal Tract losses due to turbulence, vorticity, viscous layers, heat losses, etc. Moreover, the absorption coefficient used in the simulation is not frequency dependent, which is unrealistic.

Table 2 shows the comparison between the simulated and measured formant frequencies, and their relative difference. The numerical results match on average within 7% the experimental measurements.

**Table 2:** Formant frequencies: simulation versus experimental.

		F1			F2			F3			F4			F5	
	(num)	(exp)	%	(num)	(exp)	%	(num)	(exp)	%	(num)	(exp)	%	(num)	(exp)	%
/p 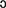 ːt/	417	482	13.49	834	833	−0.12	2,334	2,448	4.66	2,647	2,753	3.85	3,414	3,515	2.87
/st 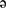 ːn/	483	523	7.65	1,134	1,043	−8.72	2,302	2,256	−2.04	2,778	2,719	−2.17	3,625	3,173	−14.25
/fuːd/	264	283	6.71	867	821	−5.60	2,122	2,193	3.24	2,736	2,774	1.37	3,361	3,464	2.97

Comparison between the experimental and numerical formant frequencies [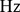] (and their relative difference) of Barnaby phonating on different vowels.

### Effect of the piriform fossae

**Figure 7:**
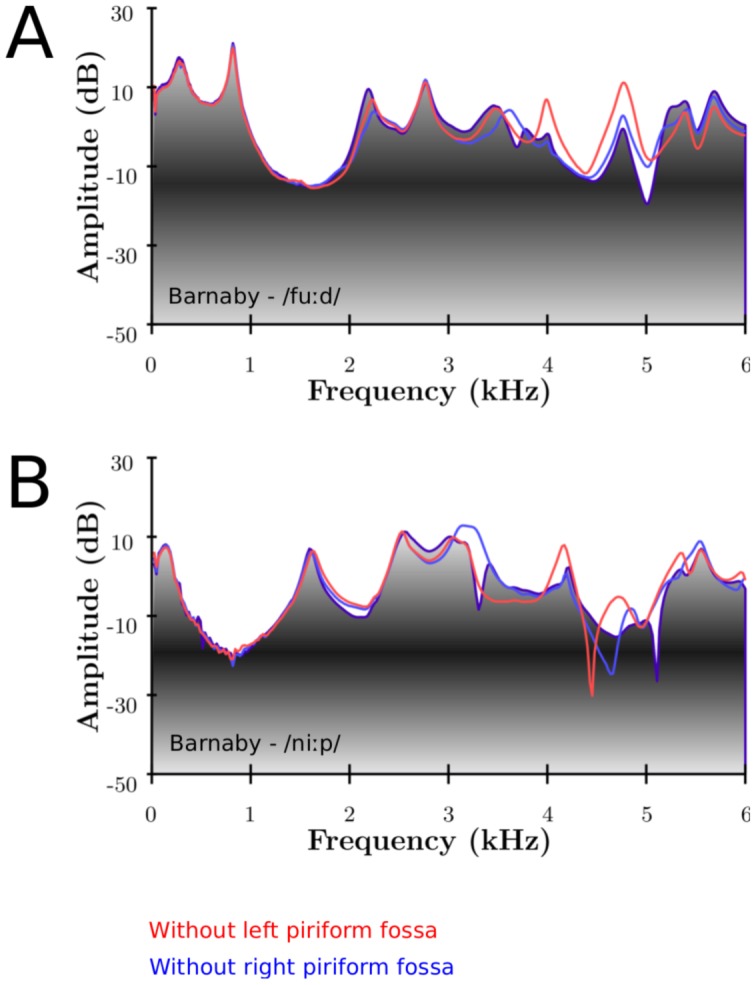
Experimental measurements of the spectral effect of the piriform fossae. Experimental measurements of MRI-based 3D-printed VT of Barnaby, singing on the vowels as in /fuːd/ (A) and /niːp/ with (greyscale) and without left (red) or right (blue) piriform fossa.

**Figure 8:**
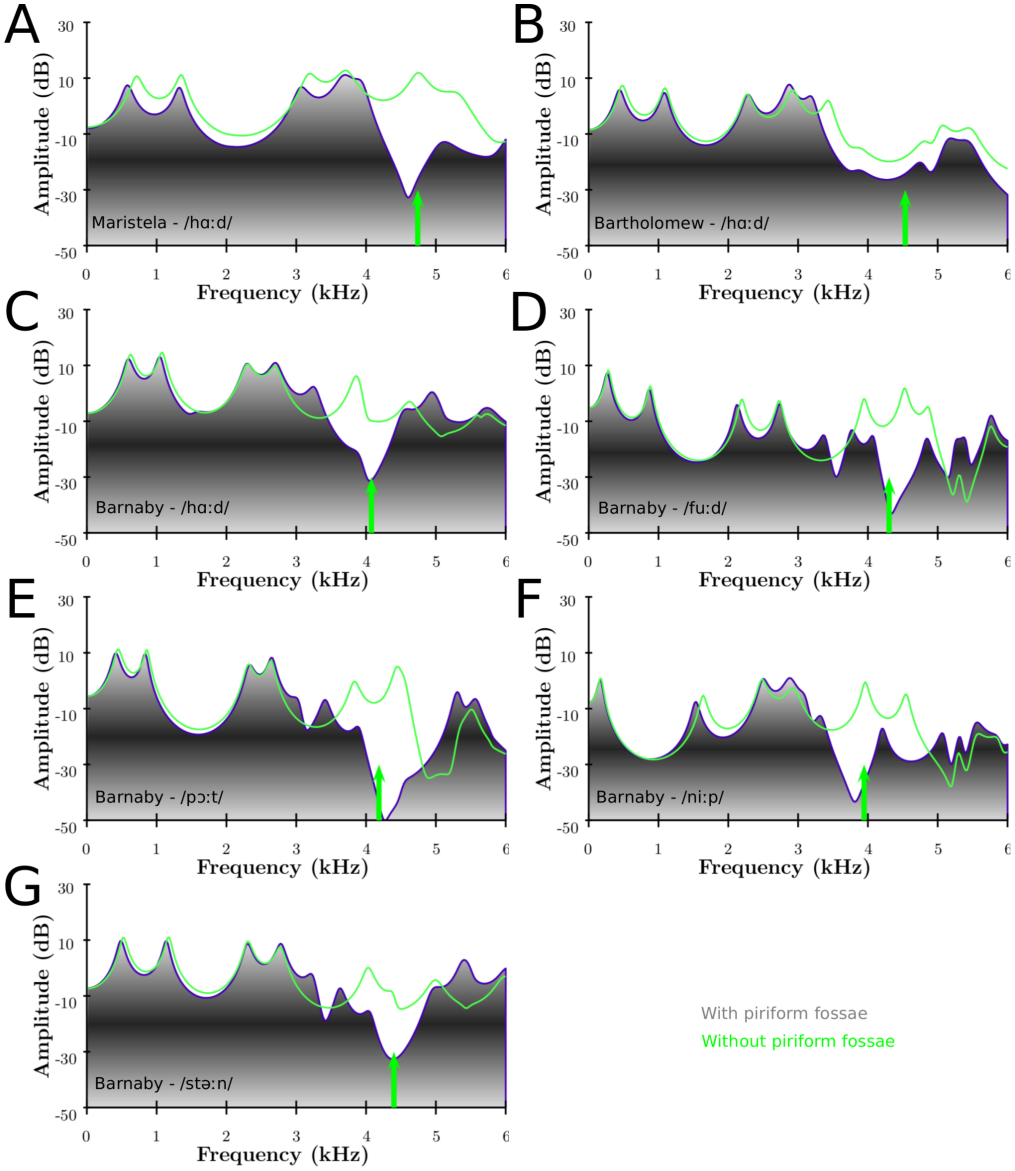
Numerical results of the spectral effect of the piriform fossae. Numerical results of MRI-based VTs with (greyscale) and without (green) piriform fossae for Maristela (A), Bartholomew (B) singing on the vowel as in /hαːd/ and Barnaby singing on the vowels as in /hαːd/ (C), /fuːd/ (D), /p
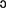
ːt/ (E), /niːp/ (F) and /st
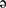
ːn/ (G). The green arrow indicates the first resonance of the fossae predicted by (16), which relates to the average length of the fossae as measured on the MRI-based VT.

Appending the piriform fossae to the main tract adds a trough around 4–5 (6) kHz in the output spectrum, probably enhancing the perception of the singer's formant cluster (SFC): a broad peak, followed by a trough. This confirms the findings of [28]. This can be seen on Fig 7 experimentally, and on Fig 8 numerically for the 3 singers. For the experimental part, the piriform fossae were filled with plasticine to simulate a vocal tract without its fossae. Since it is difficult to smooth manually the plasticine to completely fill in the piriform fossae, there are differences in observed results. The experimental results show the effect for the left and right piriform fossae individually.

Fig 7 (A, B) show the experimental results for Barnaby phonating on /fuːd/ and /niːp/ respectively (with and without piriform fossae). It can be seen that the main frequency region affected by the piriform fossae is between 4 and 5 kHz. The formants below and above this region are repelled: the formants whose frequency are lower/greater than the resonance frequency of the fossae are decreased/increased respectively. This agrees with the results found in [5]–[7], [28].

Fig 8 shows the numerical results for the 3 singers. The green arrow represents the resonance frequency of the piriform fossae derived from their length (see Table 3). Titze et al. suggested the use the quarter-wave resonator formula (eq (13) from [6]) 
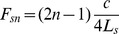
(16)where 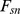 is the 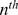 resonance of the piriform sinuses, 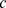 the speed of sound and 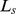 the length of the sinuses. The predicted spectral zeros are in good accordance with the numerical simulations: the longer the sinus, the lower the resonance frequency. Knowing more accurately the acoustical length of the fossae (accounting for the end correction effect) would give a more accurate prediction of the resonance frequency. The mean antiresonance frequency across the singers is 4451 Hz with a standard deviation of 340 Hz whereas the mean across the vowels of Barnaby is 4182 Hz with a standard deviation of 179 Hz. Fig 8 visually confirms the experimental results: the piriform fossae act as formants repellent, the formants with a lower/greater frequency than the resonance frequency (green arrow) see their frequency decreased/increased.

**Table 3:** Vocal Tract and piriform fossae dimensions.

	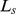	f	VTV	PV	%
Maristela - /hαːd/	17.63	4741	44.58	3.47	7.78
Bartholomew - /hαːd/	18.31	4565	37.32	1.7	4.56
Barnaby - /hαːd/	20.50	4077	80.49	1.69	2.1
Barnaby - /p 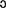 ːt/	19.97	4185	65.61	3.64	5.55
Barnaby - /st 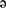 ːn/	19.00	4399	65.29	3.43	5.25
Barnaby - /fuːd/	19.42	4304	57.67	4.79	8.31
Barnaby - /niːp/	21.18	3946	65.48	3.58	5.47

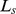
 is the length of the piriform sinuses [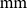], f, the antiresonance frequency generated by the piriform sinuses [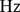], VTV is the volume of the Vocal Tract [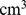], PV is the volume of the piriform fossae [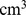], % is the ratio PV/VTV expressed in percentage.

**Figure 9:**
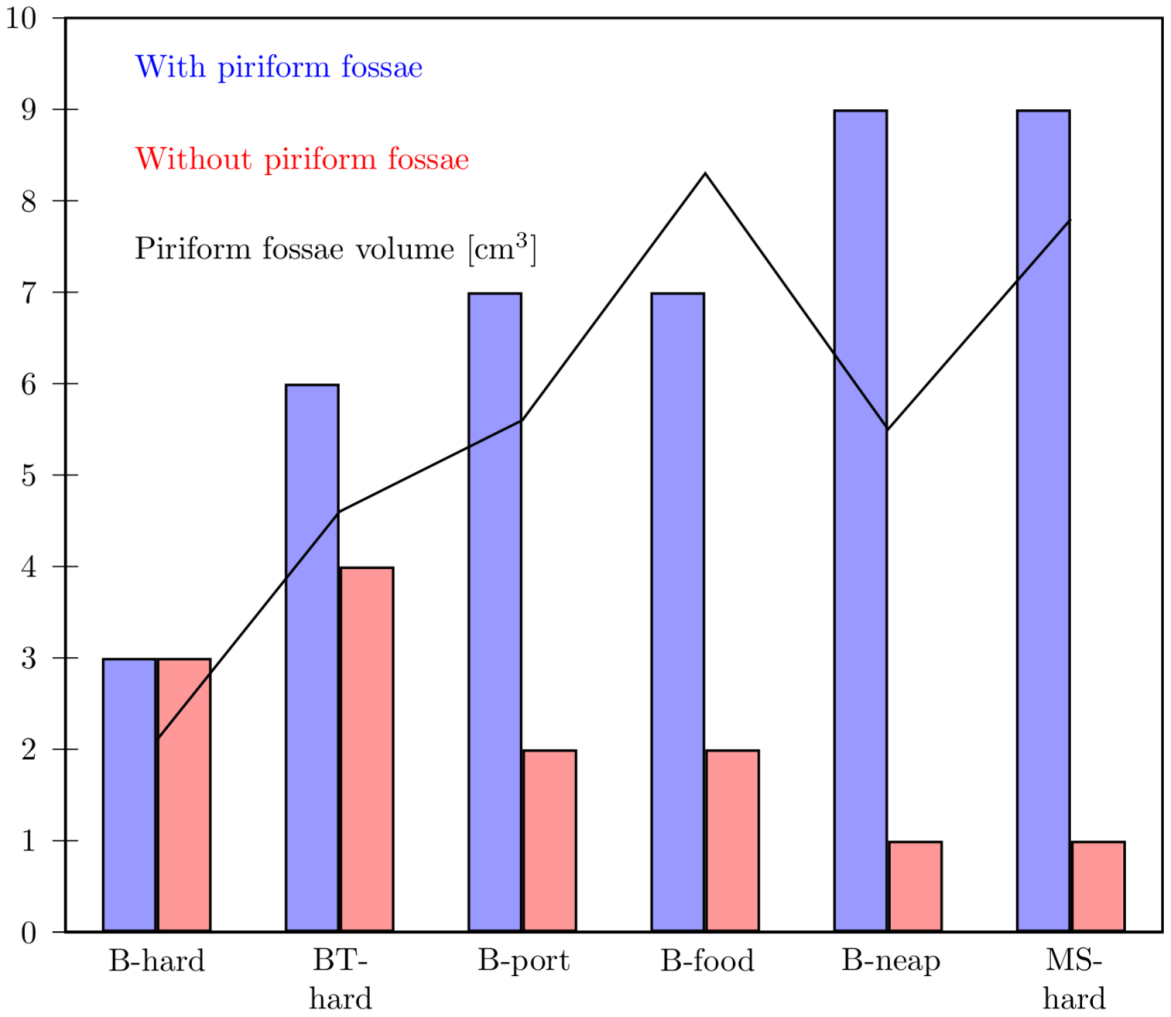
Listening test - perceptual effect of the piriform fossae. Listening test to assess perceptually the spectral effect of appending the piriform fossae to the main tract. 10 expert listeners were asked to choose between each pair of sound which one they were qualifying as a “resonant voice” [21]. The vertical bars represent the number of positive answers (up to 10) for the sound sample with (blue) and without (red) piriform fossae respectively. The volume of the piriform fossae is plotted in black. B stands for Barnaby, BT for Bartholomew and MS for Maristela.

A listening test was performed to assess perceptually the spectral impact of appending the piriform fossae to the main tract. A group of 10 expert listeners were asked to choose for each of 6 pairs of sounds which one they would qualify as being a resonant voice. One of the voice samples included the spectral effect of the piriform fossae and the other did not. The results are shown on Fig 9 where it appears that the bigger the volume of the piriform fossae, the more resonant the voice sounds, perceptually. This supports the fact that the piriform fossae spectrally enhance the perception of the SFC.

It is interesting to note that the ratio of the volume of the piriform fossae and the Vocal Tract (penultimate column in Table 3) is related to the amplitude of their effect on the spectrum: the bigger the fraction, the bigger the impact on the transfer function. See for instance Maristela, whose piriform fossae constitute 8% of the Vocal Tract volume: her piriform fossae have a relatively larger spectral impact than those of the other singers.

From Fig 8, it can be seen that the female voice tends to show a spectral trough due to the piriform fossae at a higher frequency range (around 4–5 to 6 kHz) than males (around 3.5 to 5 kHz), which is consistent with the fact that the spectral role of the piriform fossae is to emphasise the SFC.

Moreover, the physiological role of the piriform fossae is to serve as side branches to “capture” foreign bodies, instead of swallowing them, but also a part of the food (at least temporarily) and the mucous, for instance when one has a cold [29]. We suggest, therefore, that singers with large piriform fossae would be more affected than others in the production of a “resonant voice” when they have a cold or when they have just eaten certain foods which would obstruct the fossae.

## Discussion

In this study, we investigated the spectral impact of the piriform fossae on the human singing voice, on MRI-based Vocal Tracts, both experimentally (3D printed VTs) and numerically. We have introduced a new experimental method based on exponential sine sweep used in room acoustics [11], enabling transducer-independent measurements of the transfer function of 3D printed Vocal Tracts with and without piriform fossae (by mean of filling the cavities in the 3D printed models with plasticine). The transfer functions of MRI-based Vocal Tracts of 3 professional singers were simulated numerically with and without the piriform fossae.

The results support the findings previously highlighted in the literature [5]–[7], [28]: the piriform fossae create a spectral trough in the region 4–5 kHz and act as a formant repellent, i.e. appending the piriform fossae repels the formant frequencies from the antiresonance they create. Here, we have provided new data and a new measurement method to confirm this effect through numerical modelling and experimental measurements on complete (rather than half as previously reported [7]) 3D-printed MRI-based Vocal tracts and relate it to MRI-based measurements of 3 professional singers.

The plots clearly show that the SFC is spectrally emphasised by appending the piriform fossae: they act as side branches and create an antiresonance (determined by the length of the fossae, see the green lines in Fig 8) at a higher frequency than the SFC (about 1–2 kHz above the SFC). The result is that the SFC is acoustically perceived as being enhanced, as confirmed with the listening test of Fig 9. Our data indicates differences with gender: female voices tend to have the spectral trough higher than males (5–6 kHz in comparison with 4–5 kHz).

From the evolutionary standpoint, the human pharynx is divided into 2 airways, the nasopharynx and the oropharynx [9]: the piriform fossae act as the last step in the process of swallowing: they allow temporary storage of bolus of food and/or liquid to use the airway both for breathing and feeding. The dimensions of these cavities in relation to those of the epilarynx play a particular role in spectrally enhancing the SFC. In addition, our results showed that the bigger the ratio of the volume of the piriform fossae to the volume of the Vocal Tract, the bigger spectral effect they have on the transfer function. This suggests that singers with large piriform fossae might experience a larger spectral change in their singing voice when they have a cold or when they have ingested certain food which obstructs the fossae.

In the future, the listening test should include same samples comparison to benchmark and support the results. A more extensive study needs to be performed on a larger number of singers to assess how the dimensions (especially the length) of the piriform fossae defines the precise location of the spectral trough and the full extent to which singers might or might not be affected by a cold while singing.

## Supporting Information

Figure S1 **Linearisation and speaker-independence.** Convolution of the system response with the inverse filter signal. As a result, the Linear Impulse Response of the system is split from its harmonic distortions (A). The Linear impulse response is temporally separated form the harmonic distortions (B). Resonances of one cylinder opened at one end, closed at the other end, with two different transducers (C). Transfer functions of the Linear Impulse Response (LIR) in red and the harmonic distortions in blue (D).
(EPS)

Figure S2
**A smooth start/end and FFT algorithm.** The Exponential Sine Sweep 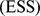 in (1) is provided with a smooth start (A) and a smooth end (B) to remove the pre- and post-ringing (See Fig 4). Algorithm used to obtain the transfer function out of an impulse response (C).(EPS)
